# Salvage Brachytherapy for Biochemically Recurrent Prostate Cancer following Primary Brachytherapy

**DOI:** 10.1155/2016/9561494

**Published:** 2016-03-22

**Authors:** John M. Lacy, William A. Wilson, Raevti Bole, Li Chen, Ali S. Meigooni, Randall G. Rowland, William H. St. Clair

**Affiliations:** ^1^Department of Urology, University of Kentucky College of Medicine, Lexington, KY 40536, USA; ^2^Department of Radiation Oncology, University of Kentucky College of Medicine, Lexington, KY 40536, USA; ^3^University of Kentucky College of Medicine, Lexington, KY 40536, USA; ^4^Department of Biostatistics, University of Kentucky College of Public Health, Lexington, KY 40536, USA; ^5^Comprehensive Cancer Centers of Nevada, Las Vegas, NV 89169, USA

## Abstract

*Purpose*. In this study, we evaluated our experience with salvage brachytherapy after discovery of biochemical recurrence after a prior brachytherapy procedure.* Methods and Materials*. From 2001 through 2012 twenty-one patients treated by brachytherapy within University of Kentucky or from outside centers developed biochemical failure and had no evidence of metastases. Computed tomography (CT) scans were evaluated; patients who had an underseeded portion of their prostate were considered for reimplantation.* Results*. The majority of the patients in this study (61.9%) were low risk and median presalvage PSA was 3.49 (range 17.41–1.68). Mean follow-up was 61 months. At last follow-up after reseeding, 11/21 (52.4%) were free of biochemical recurrence. There was a trend towards decreased freedom from biochemical recurrence in low risk patients (*p* = 0.12). International Prostate Symptom Scores (IPSS) increased at 3-month follow-up visits but decreased and were equivalent to baseline scores at 18 months.* Conclusions*. Salvage brachytherapy after primary brachytherapy is possible; however, in our experience the side-effect profile after the second brachytherapy procedure was higher than after the first brachytherapy procedure. In this cohort of patients we demonstrate that approximately 50% oncologic control, low risk patients appear to have better outcomes than others.

## 1. Introduction

The use of interstitial brachytherapy with permanent seed implantation is a well-established means of treating localized prostate cancer [1, 2]. Permanent interstitial brachytherapy for prostate cancer patients involves the insertion of radioactive seeds (containing ^125^I, ^103^Pd, or ^131^Cs), encased in titanium shells, into the prostate gland. Benefits of this treatment modality include a single procedure rather than weeks of daily external beam radiation therapy (EBRT) and equivalent oncologic control for low and intermediate risk disease [1]. Other studies have shown superior quality of life regarding sexual function and urinary bother scores for prostate seed implant compared to prostatectomy [3].

Unfortunately, up to as high as 10% to 15% of men may experience prostate-specific antigen (PSA) failure in five to ten years after interstitial brachytherapy for clinically localized prostate cancer [2, 4]. Some of these men will harbor a component of micrometastatic disease at the time of PSA failure, but a significant number will have a true local-only recurrence and potentially can be cured with a salvage local therapy. Currently, there is no consensus regarding the optimal management of patients who are believed to have a local-only recurrence after prostate radiotherapy. Palliative management options include androgen deprivation therapy or expectant management. A number of salvage therapies with curative intent have also been assessed including cryotherapy, EBRT, high-intensity focused ultrasound (HIFU), brachytherapy, and radical prostatectomy [5, 6].

Data on salvage brachytherapy after primary brachytherapy is extremely limited. Much of the data in the current literature comes from patients that were included in cohorts of patients treated with primary radiation therapy with the majority of patients undergoing EBRT. To our knowledge, there are only 2 studies that evaluate salvage brachytherapy for patients who were initially treated with brachytherapy, one in 1990 [7] in the pre-PSA era and one in 2003 [8]. In this single center review of 21 patients, we describe our experience with salvage brachytherapy for prostate cancer patients with biochemical recurrence after primary brachytherapy focusing on oncologic and functional outcomes.

## 2. Methods and Materials

From July 2001 until February 2012, we reviewed the records of all patients who underwent salvage reimplantation at our center. During this time frame 21 patients underwent a repeat brachytherapy procedure. Of the 21 patients in this analysis, 3 had a biopsy before the repeat brachytherapy procedure and the remainder did not. Of these 21 patients, 14 had their initial brachytherapy procedure performed at the University of Kentucky (UK). One of the patients initially treated at UK was found to have poor coverage at the one-month postimplant CT evaluation and subsequently had a planned reimplantation. The other 13 patients initially treated at UK had adequate postimplant dosimetry; years later they developed a rising PSA. The remaining 7 men in this cohort had their initial brachytherapy procedure at other institutions. One of these 7 patients underwent open brachytherapy procedure in 1984 and many years later developed a rising PSA. Thus, of the patients in this cohort 20 of 21 patients had an acceptable brachytherapy procedure and were later identified as having a biochemical failure defined by either the ASTRO criteria or Phoenix criteria [9]. D'Amico criteria were used to stratify risk category [10]. All patients who met the criteria for biochemical failure had a bone scan and a computed tomography (CT) scan of the abdomen and pelvis to rule out evidence of metastasis. The pelvic CT scan was also evaluated by radiation oncology and urology faculty to assess brachytherapy seed placement within the prostate. If both radiation oncology and urology faculty clinically agreed that a 3-dimensional volume of the prostate was underseeded from the initial brachytherapy procedure and the scans revealed no evidence of metastases, those patients were considered for a brachytherapy salvage procedure. If the prostate appeared to be well seeded with no gaps or evidence of metastasis the patient was excluded from a second brachytherapy procedure. There were no other exclusion criteria, with no restrictions on presalvage PSA or International Prostate Symptom Score (IPSS) survey results.

**Figure 1:**
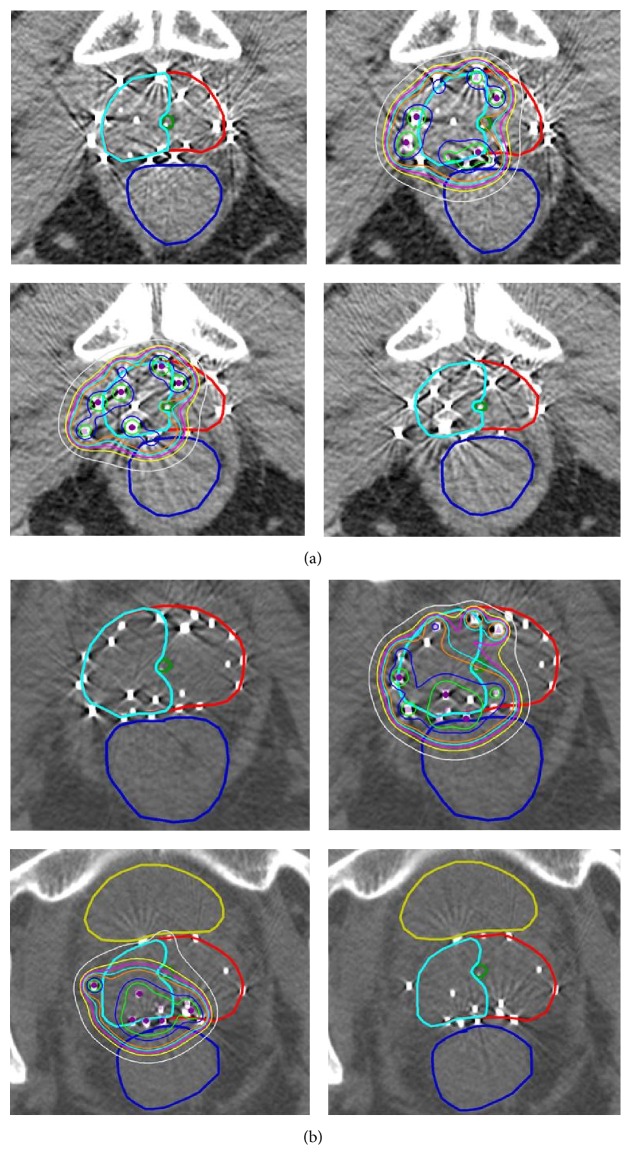
(a) Sample brachytherapy planning scans using BrachyVision software. (b) CT images showing seed distribution after reimplantation with and without reimplant isodose distributions.

A preplanning TRUS volume study was performed, and target volumes were outlined by a radiation oncologist with urology collaboration. The target volume to receive radioactive seeds only included the underseeded portion of the prostate with a small margin, of 2 mm. In addition, care was taken to keep the urethral doses low; our goal was to keep the urethral dose to no more than that of the prescribed dose. BrachyVision software was utilized for brachytherapy planning. Sample planning scans are shown in Figure 1(a). All patients were treated using ^125^I seeds, and the prescribed dose ranged from 108 Gy to 144 Gy (Table 1). When the dosimetric plan was completed, stranded seeds were purchased from a vendor (IsoAid) and the patient returned for implant in approximately 2 weeks.

Epidural anesthesia was administered and patients were placed in the dorsal lithotomy position. A urethral catheter was inserted and instilled with ultrasonic contrast (surgical lubricant with air bubbles) to visualize the urethra. A TRUS probe was placed into the rectum, and the ultrasound images were matched to the preplanned images acquired in the same manner. When the real-time images of the prostate matched the preplan images, the needles carrying the radioactive seeds were inserted and the seeds were placed. Representative postoperative CT images showing seed distribution after reimplantation with and without reimplant isodose distributions are shown in Figure 1(b).

Androgen deprivation therapy (ADT) was concurrently administered at the discretion of the treating physician. Three of 21 (14.3%) patients received one 3-month injection of Lupron along with their salvage brachytherapy. Two of these patients ultimately had biochemical failure after their reseeding.

Patients were followed up in a multidisciplinary urologic oncology clinic by both urology and radiation oncology faculty. PSA values were obtained and patients were questioned regarding potential treatment related toxicity. Lower urinary tract symptoms were evaluated and categorized by their IPSS, which were self-reported by the patients immediately prior to each appointment. Patients were also questioned about gastrointestinal complaints and other concerns and scored according to the RTOG acute and late toxicity criteria.

Summary statistics such as median and range values were calculated for patient characteristics and disease outcomes. Kaplan-Meier curves and Wilcoxon tests were used to evaluate the association between time to biomedical failure and patient categorical characteristics. Paired two-sample *t*-tests were used to determine the statistical significance of differences in a continuous variable between two different measurement times. Fisher's exact tests were used to determine the statistical significance of differences in binary outcomes between two patient groups. Significance was considered using a two-sided *p* value <0.05. Statistics were carried out using Microsoft Excel, SAS version 9.2, and R version 3.0.1.

## 3. Results

Radiation dosing utilized for the salvage therapy in this study is summarized in Table 1. At initial diagnosis of patients in this study, 3 of 21 (14.3%) patients received EBRT with their initial brachytherapy. One of 21 (4.7%) patients underwent open brachytherapy seed placement as his initial brachytherapy. Risk categories were determined based on initial PSA, Gleason score, and initial clinical stage. Thirteen of 21 (61.3%) patients were low risk, and 6 of 21 (28.6%) patients were intermediate risk; 7of 21 (33.3%) patients had initial brachytherapy at other institutions and had incomplete information to adequately calculate their risk category.

Mean presalvage PSA was 3.49 (median 3.6, range 17.41–1.68). PSA dynamics for individual patients during the study are shown in Table 2. Mean follow-up was 61 months. Median nadir was 0.7 ng/mL (range 2.97–0.01 ng/mL) and median time to nadir was 15 months. All men undergoing a salvage brachytherapy procedure demonstrated an initial decline in their serum PSA. Median time to biochemical failure (according to the Phoenix criteria) of the men failing the second implant was 25 months (range 11–71 months).

### 3.1. Toxicity

A few patients experienced adverse outcomes following salvage brachytherapy during our study period. Two patients experienced Clavien grade I urinary incontinence; one patient experienced each of the following: Clavien grade IIIb bladder neck contracture, rectourethral fistula, and leiomyosarcoma.

**Figure 2:**
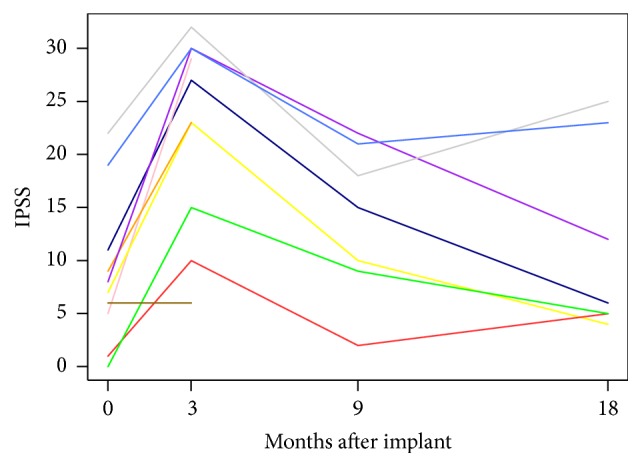
IPSS scores throughout follow-up after salvage brachytherapy.

Figure 2 depicts urinary tract toxicity obtained from IPSS questionnaires. As expected, IPSS scores increased from a median of 7 to a median of 23 at 3 months following therapy (*p* < 0.0001). Median IPSS dropped to 11 at 9 months (*p* = 0.0005). IPSS continued to decrease at 18 months with a median of 5, which was not significantly different from baseline (*p* = 0.294).

Two of 21 (9.6%) patients developed de novo urinary incontinence, and 1 of 21 (4.8%) developed a rectourethral fistula. One of 21 (4.8%) patients developed bladder outlet obstruction secondary to fibrosis, requiring endoscopic correction (transurethral incision of bladder neck). One patient developed a leiomyosarcoma that ultimately required cystoprostatectomy but had biochemical control of prostate cancer.

Table 3 summarizes changes in sexual function during the study. Eleven of 21 (52.3%) patients had sufficient data to compare pre- and posttherapy sexual function. Six of 11 (54.5%) patients had stable sexual function, and 5 of 11 (45.5%) had decreased sexual function.

### 3.2. Time to Failure

**Figure 3:**
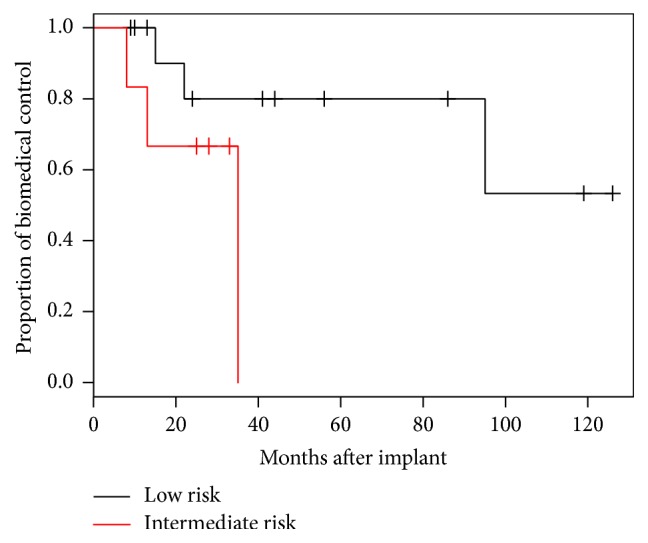
Effect of initial risk category on risk of biochemical recurrence.

The mean decrease in PSA was 4.5 ng/dL (median 3.4, range 0.3–15.0) after salvage brachytherapy. At the time of analysis, 11 of 21 (52.4%) patients had not experienced biochemical recurrence with a mean follow-up of 61 months.  Figure 3 shows a Kaplan-Meier curve for time to biochemical recurrence based on initial risk category. For the men that failed a second brachytherapy procedure, the median time to failure was 25 months, with a range of 11 to 71 months. There was a trend towards increased time to biochemical failure in the low risk group compared to the intermediate risk group (47 months versus 27 months, *p* = 0.12).

## 4. Discussion

Prostate cancer patients who experience biochemical failure after initial radiotherapy have a number of options for subsequent treatment: observation, androgen deprivation therapy, salvage radical prostatectomy, salvage cryotherapy, high-intensity focused ultrasound (HIFU), and additional radiotherapy. Salvage therapy is extremely important to control locally recurrent disease and prevent metastases, as lack of further treatment after biochemical recurrence is correlated with the development of clinical disease within 5 years in up to 75% of patients [11].

Biochemical disease-free survival rates of 55–61% at 5-year follow-up have been reported with salvage radical prostatectomy. These were relatively small series with 42, 100, and 138 patients [12–14]. Surgical intervention is even able to achieve a 51% disease-free survival rate at 5-year follow-up in patients with a Gleason score of 8 or higher [15, 16]. However, there are a number of drawbacks to this technique as well. It can be a technically challenging procedure due to residual irradiated tissue damage with increased risk of a number of major complications. Up to 40% of patients are afflicted with urinary incontinence and 25% suffer from bladder neck stricture following this intervention [17]. The risk of rectal injury is 2–9% and urinary fistula is <4% [18].

On the other hand, salvage cryotherapy after initial radiation is a minimally invasive option with improved efficacy, especially following the development of modern techniques to combat issues such as incomplete freezing of tissue. One study reported biochemical disease-free survival, defined as PSA <0.5 ng/mL, at 73% for low risk patients, 45% for intermediate risk patients, and 11% for high risk patients after a median 33.5-months follow-up and actuarial projection of 60 months [19]. Salvage cryotherapy may be a less challenging procedure to perform than salvage radical prostatectomy, giving it minimal variation in outcome across health providers [20]. In addition, the reported rates of complication are low enough to make it an attractive option for older patients, with urinary incontinence affecting less than 10% of patients [20, 21]. Cryosurgical ablation of the whole prostate gland has also been strongly associated with impotence. Focal cryoablation techniques may limit this adverse effect by relying on targeted image-guided biopsy to guide therapy towards undertreated areas or areas of recurrence, decreasing morbidity [22].

Salvage HIFU has also been studied as a treatment for locally recurrent prostate cancer following external beam radiation therapy (EBRT) only. In a study of 167 European patients, the biochemical disease-free survival was 53%, 42%, and 25% for low, intermediate, and high risk patients, respectively [23]. While the rate of metastatic disease development in this cohort of patients was comparable to that seen in patients treated with other salvage therapies, the complication rate was remarkably low, especially with regard to urinary incontinence and bladder outlet obstruction. Another European study showed that the rate of bladder outlet obstruction decreased from 30% to 15% while the rate of surgical intervention for urinary incontinence decreased from 15% to 5% [24]. Salvage HIFU, however, is not recommended for patients who had initial brachytherapy due to the reflective capacity of the implants, which can redirect excess energy onto surrounding structure such as the rectum and urethra [25].

In recent years, single center brachytherapy studies have shown up to 75% biochemical disease-free survival at 4 years with permanent brachytherapy [17, 26]. In low risk patients who underwent EBRT alone, one recent study showed a biochemical disease control percentage of 85.6% after 5 years [27]. High dose rate (HDR) brachytherapy is now being used as a more customizable form of dosage for patients [28, 29]. However, these doses could cause further toxicity to irradiated damaged tissue and were found in two studies to cause grade 2 urethral strictures in 71% of patients [28, 30]. One prospective study that used MRI-guided seed placement rather than TRUS guidance found that this method was able to keep the incidence of gastrointestinal and genitourinary toxicity requiring surgical treatment at 15% in 4 years [17]. They also found that toxicity was decreased in men who had longer than 4.5-year interval between radiation therapies.

Data on salvage brachytherapy following primary brachytherapy, however, is limited. Studies usually mix patients that were treated with primary brachytherapy with those who initially underwent EBRT. One study from 1990 reported outcomes of salvage brachytherapy seed implantation in 13 patients after initial brachytherapy treatment [31]. Recurrence in this study was detected by digital rectal examination and confirmed with prostate biopsy. A study by Grimm et al. [32] showed that of 31 patients who were reimplanted with seeds for salvage brachytherapy after initial brachytherapy, 87% had biochemical disease control at 31 months using the ASTRO criteria. Interestingly, all but one of these patients underwent 3 months of ADT at the time of salvage brachytherapy. Twenty of 31 (64.5%) of these patients had local recurrence within the prostate, and 11of 31 (35.5%) had disease within the seminal vesicles. Eleven of 31 (35.5%) patients were treated with salvage therapy within 24 months of their primary therapy, and 20 of 31 (64.5%) were treated more than 24 months after their initial therapy. In light of these results, treatment of local recurrence of prostate cancer after initial brachytherapy is an area of evolving interest.

In our series, we show 52.4% freedom from biochemical recurrence with a mean follow-up of 61 months. These data are not as efficacious as those in the Koutrouvelis series, perhaps due to the longer follow-up in our series. It is also possible that 3 months of ADT with salvage brachytherapy led to their superior results, though, in our small subset of patients who received ADT with their salvage therapy, two-thirds of patients had biochemical recurrence during our study. Alternatively, this finding in our study could be due to a selection bias by the treating physician based on the individual patients' risk factors.

When it comes to selecting any salvage therapy modality, accurately characterizing the presence, location, and extent of cancer recurrence in an individual is paramount. Improved cancer targeting and staging techniques significantly improve the risk-benefit ratio for patients with low and intermediate risk disease by allowing physicians to remove malignancy while preserving as much normal tissue and anatomy as possible [33]. In one study, however, the widely used 12-core TRUS biopsy technique was only able to predict unilateral prostate cancer in less than 30% of cases, making it less effective at choosing patients for focal therapy [34]. Instead, using a transperineal template-guided mapping biopsy (TTMB) technique provides better access to the apical and anterior portions of the prostate where up to one-third of significant cancer is located [35, 36]. A recent study used TTMB with multiparametric MRI to assess their combined ability to detect clinically significant cancer, defined as Gleason 6 with tumor length over 3 mm and any Gleason 7 and above [37]. The result of the combined testing was a positive predictive value of 83% and negative predictive value of 91%, which reliably demonstrates the ability to rule out clinically significant prostate cancer [37]. In light of a rising PSA, the negative predictive value of these additional criteria would be helpful in focusing salvage therapy towards patients with clinically significant disease [38].

Alternatively, metabolic imaging offers a less invasive method of detection of both localized and systemic tumor burden. The available literature varies with regard to the optimal PSA value at which to initiate ^18^F-Choline PET/CT imaging, but there appears to be a strong correlation between increasing PSA values and the positive predictive ability of this tool [39]. In one study of 250 prostate cancer patients with biochemical recurrence, ^18^F-Choline PET/CT showed 77% sensitivity for cancer detection at a PSA level greater than 0.3 ng/mL and had a particularly high sensitivity in patients treated with ADT as compared to those who did not receive ADT [40].

Our study has several key limitations that warrant discussion. It is a retrospective, nonrandomized study with inherent selection and treatment biases. Patients were treated with salvage therapy based on biochemical recurrence alone, analogous to the delivery of salvage external radiation therapy for a recurrence following a radical prostatectomy. Therefore, the majority of patients did not undergo TTMB or repeat TRUS biopsy prior to salvage therapy, leaving the possibility that their pathology was different from that of their original prostate biopsy and skewing their presalvage risk stratification. Follow-up was not standardized, leading to incomplete data on erectile function, lower urinary tract symptoms, and GI toxicity. Finally, while all patients underwent primary brachytherapy prior to their salvage brachytherapy, the cohort remains somewhat heterogeneous. Three of 21 (14.3%) patients underwent EBRT with their primary brachytherapy. Seven of 21 (33.3%) patients had ADT with primary therapy and 3 of 21 (14.3%) had ADT with their salvage brachytherapy. One of 21 (4.7%) patients had open brachytherapy seed placement as initial treatment.

Salvage brachytherapy is an intriguing treatment option for patients with biochemically recurrent prostate cancer who underwent primary brachytherapy. In our study, this modality appears to provide adequate prostate cancer control in select men with underseeded areas on cross-sectional imaging. There is a trend towards decreased efficacy of salvage brachytherapy in patients who were intermediate risk at initial presentation. Side effects of treatment are higher than expected with a single brachytherapy implantation; out of 21 patients 3 individuals developed grade 3 toxicities. The majority of lower urinary tract symptoms resolve within 9 months of treatment and minimal gastrointestinal side effects. Further studies are warranted to compare this treatment modality to other salvage therapies in patients who underwent primary brachytherapy. Further studies would perhaps be made more meaningful by utilizing more advanced methods to evaluate for location of the recurrence within or beyond the prostate. Technologies have emerged which may be beneficial in better selecting patients for salvage brachytherapy. For example, methods such as transperineal mapping, multiparametric MRI, and/or ^18^F-Choline PET/CT scans may make partial prostate implants more successful by better localization of the recurrent disease. However, we are unaware of any of these new technologies being used to select patients for salvage prostate brachytherapy. Metabolic imaging using PET/CT likely will increase the detection of metastatic prostate cancer [41] thereby better selecting a population that could benefit from the added brachytherapy procedure.

## Figures and Tables

**Table 1:** Radiation dosing.

Patient #	Age at initial treatment (yr)	Prostate cancer risk strata^@^	ADT with BRT #1	ADT with BRT #2	Initial dose	Time b/w Rx (mo)	Salvage dose	Volume treated (cc)
1^≠^	57	Low	No	No	115 Gy	38	144 Gy	21.55
2	61	Low	No	No	144 Gy	32	144 Gy	13.23
3	65	Low	No	Yes	144 Gy	26	144 Gy	7.56
4	63	Intermediate	No	No	144 Gy	34	144 Gy	9.98
5	59	Low	No	No	144 Gy	26	108 Gy	21.58
6^≠^	50	Low	No	Yes	115 Gy	67	108 Gy	26.66
7	48	x	No	No	x	287	108 Gy	21.31
8	60	Low	No	No	144 Gy	123	108 Gy	6.84
9	55	Low	Yes	No	144 Gy	120	108 Gy	7.76
10	57	Low	No	No	144 Gy	19	120 Gy	23.78
11	48	Low	Yes	No	144 Gy	25	125 Gy	16.03
12	66	Low	No	No	144 Gy	46	108 Gy	18.16
13	44	Low	No	No	144 Gy	31	144 Gy	12.05
14	50	Intermediate	No	No	144 Gy	87	144 Gy	15.51
15^*∗*^	66	Intermediate	Yes	No	108 Gy	42	108 Gy	8.1
16	71	Intermediate	No	No	144 Gy	45	108 Gy	29.36
17^*∗*^	63	Intermediate	No	Yes	108 Gy	48	108 Gy	9.74
18^*∗∗*^	42	x	Yes	No	x	50	120 Gy	12.94
19^*∗*^	58	Intermediate	Yes	No	108 Gy	105	108 Gy	8.67
20	59	Low	Yes	No	144 Gy	48	144 Gy	27.31
21	72	Low	Yes	No	140 Gy	21	140 Gy	19.93

^*∗*^Patients who underwent initial external beam radiation therapy.

^*∗∗*^Patients who underwent initial open brachytherapy.

^≠^Patients who had palladium-103 seeds for initial brachytherapy.

x = data point not known.

ADT = androgen deprivation therapy; BRT = brachytherapy.

@ = D'amico risk strata [10].

**Table 2:** PSA dynamics.

Patient #	Age at initial treatment (yrs)	Time between 1st and 2nd Brachytherapy (mo)	Pre-salvage PSA (ng/mL)	Presalvage PSA (ng/dL)	Follow-up (mo)	Time to nadir (mo)
1	57	38	6.3	3.6	149	21
2	61	32	5.3	3.6	132	31
3	65	26	4.5	4.4	86	73
4	63	34	10	2.5	111	6
5	59	26	4.9	2.7	119	10
6	50	67	8	14.9	83	10
7	48	287	x	8.6	34	22
8	60	123	7.2	17.41	10	9
9	55	120	6.3	1.53	67	7
10	57	19	5.3	3.49	49	48
11	48	25	6.93	2.77	41	41
12	66	4	4.7	1.86	15	4
13	44	31	8.7	2.41	41	29
14	50	87	7.4	7.3	39	33
15	66	42	19.1	1.68	52	9
16	71	45	6.4	3.11	40	5
17	63	48	8.2	13.8	49	3
18	42	50	1	0.98	40	41
19	58	105	x	11.58	52	17
20	59	48	5.7	4.24	25	7
21	72	21	5.8	3.1	21	15

Mean	**57.8**	**62.9**	**6.9**	**5.5**	**61.4**	**20.9**
Median	**59.0**	**45.0**	**6.3**	**3.5**	**49.0**	**15.0**
SD	**8.4**	**60.2**	**3.5**	**4.9**	**37.9**	**18.3**
*n*	**21**	**21**	**19**	**21**	**21**	**21**

x = data point not known.

**Table 3:** Erectile function.

Patient #	Age (yrs)	Baseline ED	18 mo f/u
1	57	1	2
2	61	1	2
3	65	1	3
4	63	1	2
5	59	2	x
6	50	3	x
7	48	1	1
8	60	1	2
9	55	3	x
10	57	x	x
11	48	x	x
12	66	2	2
13	44	2	2
14	50	3	3
15	66	x	2
16	71	2	x
17	63	3	x
18	42	3	3
19	58	2	2
20	59	3	x
21	72	1	x

1 = no difficulty with erection.

2 = erectile function, but not significant enough for penetration.

3 = impotent.

x represents missing data.

## Abbreviations

CT: Computed tomography scan
PSA: Prostate-specific antigen
TRUS: Transrectal ultrasound
IPSS: International Prostate Symptom Score
EBRT: External beam radiation therapy
HIFU: High-intensity focused ultrasound
ADT: Androgen deprivation therapy
MRI: Magnetic resonance imaging
HDR: High dose rate
GI: Gastrointestinal
GU: Genitourinary
TTMB: Transperineal template-guided mapping biopsy.

## Additional Points

In this project, the authors describe the eleven years of experiences at a single institution of salvage therapy in prostate cancer patients with biochemical failure and no evidence of metastatic disease after interstitial brachytherapy. Images from the CT scans were evaluated by radiation oncology and urology faculty. Patients with underseeded areas on the CT images were considered for reimplantation with I-125 interstitial brachytherapy; 21 patients were reimplanted. Our results indicated that the salvage brachytherapy to underseeded areas after primary brachytherapy in patients with biochemical recurrence can be a successful procedure.

## References

[1] Arvold N. D., Chen M.-H., Moul J. W. (2011). Risk of death from prostate cancer after radical prostatectomy or brachytherapy in men with low or intermediate risk disease. *Journal of Urology*.

[2] Vassil A. D., Murphy E. S., Reddy C. A. (2010). Five year biochemical recurrence free survival for intermediate risk prostate cancer after radical prostatectomy, external beam radiation therapy or permanent seed implantation. *Urology*.

[3] Malcolm J. B., Fabrizio M. D., Barone B. B. (2010). Quality of life after open or robotic prostatectomy, cryoablation or brachytherapy for localized prostate cancer. *The Journal of Urology*.

[4] Koukourakis G., Kelekis N., Armonis V., Kouloulias V. (2009). Brachytherapy for prostate cancer: a systematic review. *Advances in Urology*.

[5] Shekarriz B., Upadhyay J., Pontes J. E. (2001). Salvage radical prostatectomy. *Urologic Clinics of North America*.

[6] Russo P. (2000). Salvage radical prostatectomy after radiation therapy and brachytherapy. *Journal of Endourology*.

[7] Wallner K. E., Nori D., Morse M. J., Sogani P. C., Whitmore W. F., Fuks Z. (1990). 125Iodine reimplantation for locally progressive prostatic carcinoma. *Journal of Urology*.

[8] Koutrouvelis P., Hendricks F., Lailas N. (2003). Salvage reimplantation in patient with local recurrent prostate carcinoma after brachytherapy with three dimensional computed tomography-guided permanent pararectal implant. *Technology in Cancer Research and Treatment*.

[9] Roach M., Hanks G., Thames H. (2006). Defining biochemical failure following radiotherapy with or without hormonal therapy in men with clinically localized prostate cancer: recommendations of the RTOG-ASTRO Phoenix Consensus Conference. *International Journal of Radiation Oncology, Biology, Physics*.

[10] D'Amico A. V., Whittington R., Bruce Malkowicz S. (1998). Biochemical outcome after radical prostatectomy, external beam radiation therapy, or interstitial radiation therapy for clinically localized prostate cancer. *Journal of the American Medical Association*.

[11] Lee W. R., Hanks G. E., Hanlon A. (1997). Increasing prostate-specific antigen profile following definitive radiation therapy for localized prostate cancer: clinical observations. *Journal of Clinical Oncology*.

[12] Bianco F. J., Scardino P. T., Stephenson A. J., DiBlasio C. J., Fearn P. A., Eastham J. A. (2005). Long-term oncologic results of salvage radical prostatectomy for locally recurrent prostate cancer after radiotherapy. *International Journal of Radiation Oncology, Biology, Physics*.

[13] Ward J. F., Sebo T. J., Blute M. L., Zincke H. (2005). Salvage surgery for radiorecurrent prostate cancer: contemporary outcomes. *Journal of Urology*.

[14] Pisters L. L., Leibovici D., Blute M. (2009). Locally recurrent prostate cancer after initial radiation therapy: a comparison of salvage radical prostatectomy versus cryotherapy. *The Journal of Urology*.

[15] Stephenson A. J., Eastham J. A. (2005). Role of salvage radical prostatectomy for recurrent prostate cancer after radiation therapy. *Journal of Clinical Oncology*.

[16] Chade D. C., Shariat S. F., Cronin A. M. (2011). Salvage radical prostatectomy for radiation-recurrent prostate cancer: a multi-institutional collaboration. *European Urology*.

[17] Nguyen P. L., Chen M.-H., D'Amico A. V. (2007). Magnetic resonance image-guided salvage brachytherapy after radiation in select men who initially presented with favorable-risk prostate cancer: a prospective phase 2 study. *Cancer*.

[18] Pisters L. L., Ward J. F. (2014). Salvage therapy after primary non-surgical therapy for prostate cancer. *AUA Update Series*.

[19] Ismail M., Ahmed S., Kastner C., Davies J. (2007). Salvage cryotherapy for recurrent prostate cancer after radiation failure: a prospective case series of the first 100 patients. *BJU International*.

[20] Pisters L. L., Rewcastle J. C., Donnelly B. J., Lugnani F. M., Katz A. E., Jones J. S. (2008). Salvage prostate cryoablation: initial results from the cryo on-line data registry. *Journal of Urology*.

[21] Galosi A. B., Lugnani F., Muzzonigro G. (2007). Salvage cryosurgery for recurrent prostate carcinoma after radiotherapy. *Journal of Endourology*.

[22] Kanthabalan A., Arya M., Punwani S. (2013). Role of focal salvage ablative therapy in localised radiorecurrent prostate cancer. *World Journal of Urology*.

[23] Murat F.-J., Poissonnier L., Rabilloud M. (2009). Mid-term results demonstrate salvage high-intensity focused ultrasound (HIFU) as an effective and acceptably morbid salvage treatment option for locally radiorecurrent prostate cancer. *European Urology*.

[24] Crouzet S., Brown S., Berge V. Multicentric oncologic outcomes of salvage HIFU for local failure after external beam radio therapy: 7 years biochemical survival of 929 patients.

[25] Ahmed H. U., Ishaq A., Zacharakis E. (2009). Rectal fistulae after salvage high-intensity focused ultrasound for recurrent prostate cancer after combined brachytherapy and external beam radiotherapy. *BJU International*.

[26] Burri R. J., Stone N. N., Unger P., Stock R. G. (2010). Long-term outcome and toxicity of salvage brachytherapy for local failure after initial radiotherapy for prostate cancer. *International Journal of Radiation Oncology Biology Physics*.

[27] Vargas C., Swartz D., Vashi A. (2014). Salvage brachytherapy for recurrent prostate cancer. *Brachytherapy*.

[28] Lee B., Shinohara K., Weinberg V. (2007). Feasibility of high-dose-rate brachytherapy salvage for local prostate cancer recurrence after radiotherapy: the University of California-San Francisco experience. *International Journal of Radiation Oncology, Biology, Physics*.

[29] Jo Y., Fujii T., Hara R. (2012). Salvage high-dose-rate brachytherapy for local prostate cancer recurrence after radiotherapy-Preliminary results. *BJU International*.

[30] Tharp M., Hardacre M., Bennett R., Jones W. T., Stuhldreher D., Vaught J. (2008). Prostate high-dose-rate brachytherapy as salvage treatment of local failure after previous external or permanent seed irradiation for prostate cancer. *Brachytherapy*.

[31] Blasko J. C., Grimm P. D., Ragde H. (1993). Brachytherapy and organ preservation in the management of carcinoma of the prostate. *Seminars in Radiation Oncology*.

[32] Grimm P. D., Blasko J. C., Radge H. (1994). Ultrasound-guided transperineal implantation of iodine-125 and palladium-103 for the treatment of early stage prostate cancer; technical concepts in planning, operative technique and evaluation. *Atlas of the Urologic Clinics of North America*.

[33] Smith D. W., Stoimenova D., Eid K., Barqawi A. (2012). The role of targeted focal therapy in the management of low-risk prostate cancer: update on current challenges. *Prostate Cancer*.

[34] Tareen B., Godoy G., Sankin A., Temkin S., Lepor H., Taneja S. S. (2009). Can contemporary transrectal prostate biopsy accurately select candidates for hemi-ablative focal therapy of prostate cancer?. *BJU International*.

[35] Taira A. V., Merrick G. S., Galbreath R. W. (2010). Performance of transperineal template-guided mapping biopsy in detecting prostate cancer in the initial and repeat biopsy setting. *Prostate Cancer and Prostatic Diseases*.

[36] McNeal J. E., Redwine E. A., Freiha F. S., Stamey T. A. (1988). Zonal distribution of prostatic adenocarcinoma. Correlation with histologic pattern and direction of spread. *The American Journal of Surgical Pathology*.

[37] Tran M., Thompson J., Böhm M. (2016). Combination of multiparametric MRI and transperineal template-guided mapping biopsy of the prostate to identify candidates for hemi-ablative focal therapy. *BJU International*.

[38] Abd-Alazeez M., Ahmed H. U., Arya M. (2014). The accuracy of multiparametric MRI in men with negative biopsy and elevated PSA level—can it rule out clinically significant prostate cancer?. *Urologic Oncology: Seminars and Original Investigations*.

[39] Chondrogiannis S., Marzola M. C., Ferretti A. (2013). Role of ^18^F-choline PET/CT in suspicion of relapse following definitive radiotherapy for prostate cancer. *European Journal of Nuclear Medicine and Molecular Imaging*.

[40] Beheshti M., Haim S., Zakavi R. (2013). Impact of 18F-choline PET/CT in prostate cancer patients with biochemical recurrence: influence of androgen deprivation therapy and correlation with PSA kinetics. *Journal of Nuclear Medicine*.

[41] Tombal B., Lecouvet F. (2012). Modern detection of prostate cancer's bone metastasis: is the bone scan era over?. *Advances in Urology*.

